# Sudden Loss and Indicators of Resilience: A Narrative Therapy Case Study

**DOI:** 10.17505/jpor.2025.28329

**Published:** 2025-10-02

**Authors:** Thanh Tu Nguyen, Phan Thi Kim Ngoc, Huynh Thi Cam Nhung, Ho Nguyen Minh Tan, Vo Trang Nguyen Du, Trinh Thi Kieu Oanh

**Affiliations:** 1 Vietnam National University, Ho Chi Minh City, Vietnam; 2 Faculty of Psychology, University of Social Sciences and Humanities, Ho Chi Minh City, Vietnam

**Keywords:** sudden loss, grief, resilience, narrative therapy, case study

## Abstract

**Objectives:**

This case study explores how narrative therapy facilitates personalized meaning-making and emotional healing for Ann, a 57-year-old Vietnamese widow, following her loved one’s sudden death.

**Methods:**

Grounded in Ann’s Buddhist beliefs and collectivist background, the therapy provided a safe space for her to express complex emotions like guilt, sadness, and anger. Utilizing a three-stage trauma recovery model—externalization, deconstruction, and exceptional questions—helped her explore new meanings, re-author her story, and build resilience and purpose within her unique personal and cultural context.

**Results:**

Throughout the therapeutic process, the client gained awareness of her loss and reconstructed new meanings within her personal story. She recognized both the possibilities and limitations of her experiences. By the final session, she was able to confidently articulate her gratitude, commit to self-care, and actively support her community, demonstrating increased resilience and self-efficacy.

**Conclusions:**

This case illustrates how a personalized narrative approach facilitates growth by helping the client acknowledge her loss, reframe her story, and discover her voice. It emphasizes the importance of honouring individual stories and cultural contexts, fostering self-awareness, resilience, and a commitment to self-care and community, supporting ongoing healing and empowerment.

## Introduction

This case study focuses on the application of narrative therapy in a non-Western, collectivist cultural context—specifically Vietnam—to facilitate resilience in a widow coping with sudden loss. By examining the therapeutic process through a person-oriented lens, it highlights how narrative techniques foster key indicators of resilience: self-efficacy, adaptation, purpose, and resource utilization, while honoring cultural and spiritual elements.

Individuals who experience the sudden loss of loved ones require considerable strengths to embark on a journey toward healing (Bonanno, 2004). Many survivors require counselling support to navigate their grief effectively (Worden, 2018). This case study examines the journey from loss to resilience of a mother, Ann (pseudonym), as she copes with the unexpected death of a loved one. Using narrative therapy techniques, the client undergoes three phases across seven sessions. *Phase 1* focuses on establishing a safe environment for the client to express sadness, anger, and confusion while being supported by her belief in the Buddhist tradition. *Phase 2* involves exploring, recognizing, and identifying available resources. *Phase 3* acknowledges the client’s progression from loss to survival.

The primary objective of this article is to explore how narrative therapy can help survivors process their loss and find meaning in life. Utilizing techniques like externalization, deconstruction, and exceptional questions, narrative therapy offers a transformative framework to foster resilience by cultivating four key components: *self-efficacy* (confidence in coping), *adaptation* (adjusting to new realities), *purpose* (finding meaning), and *resource utilization* (leveraging supports). This study illustrates how narrative therapy can facilitate these components to promote healing, using the case of Ann, a 57-year-old Vietnamese widow. It examines the interplay of sudden loss, grief, and resilience, highlighting how narrative therapy can enable survivors to re-author loss narratives, identify exceptions to grief-dominated moments, reconnect with strengths, and balance grief with responsibilities.

### Sudden Loss and Grief: A Narrative of Disruptions

Sudden loss, such as a death from depression-related causes, elicits intense emotions like shock, guilt, and helplessness, often complicating grief due to its unexpected nature (Keyes et al., 2014). Unlike anticipated loss, it offers no preparatory period, increasing risks of prolonged distress or post-traumatic stress (O’Brien et al., 2016). Kübler-Ross’s (1973) five stages of grief—denial, anger, bargaining, depression, and acceptance—provide a framework, though individuals navigate these non-linearly (Bonanno, 2004). Grief manifests both physiologically (e.g., insomnia, fatigue) and psychologically, reflecting the interconnectedness of mind and body (Tchalova & Eisenberger, 2015; Kowalski & Bondmass, 2007).

Sudden loss fragments personal narratives, triggering existential crises and identity disruption (Neimeyer, 2001). For Ann, her husband’s death after 35 years of a loving marriage provoked guilt (“I did not take him to the hospital”), fear, loneliness, and anger, compounded by her struggle to support her children abroad. This aligns with Stroebe and Schut’s (1999) Dual Process Model, in which bereaved individuals oscillate between loss-oriented (e.g., grieving) and restoration-oriented (e.g., caregiving) coping strategies. Narrative therapy addressed this disruption by reconstructing narratives, fostering resilience through her belief in her Buddhist tradition, meaning-making and agency across self-efficacy, adaptation, purpose, and resource utilization (White & Epston, 1990).

### Narrative Therapy: Fostering Resilience

Narrative therapy is a therapeutic approach that emphasizes the stories individuals tell about their lives and how these stories form their identities and experiences. It focuses on separating the problem from the person, viewing problems as external to the individual’s essential identity, and empowering individuals to re-author their stories by highlighting their potential, values, and unique outcomes (White & Epston, 1990). Narrative therapy therefore posits that identities are shaped through stories, which sudden loss disrupts (White & Epston, 1990). It employs *externalization* to separate individuals from problems (e.g., viewing grief as external), *deconstruction* to challenge problem-saturated narratives, and *exceptional questions* to highlight moments when grief is less dominant (Freedman & Combs, 1996).

These techniques enable survivors to re-author narratives, shifting from a state of helplessness to one of resilience by strengthening the four components: *self-efficacy, adaptation, purpose,* and *resource utilization* (Morgan, 2000). In Ann’s case, externalization helped her view guilt as separate from her identity, reducing self-blame (Khasanah & Habsy, 2025). Deconstruction challenged narratives such as “I failed him,” while exceptional questions (“When did you feel less overwhelmed?”) unearthed strengths, such as her support for her children. Research supports these approaches. Carr (1998) found that externalization mitigates the intensity of grief, and Morgan (2000) notes that exceptional questions amplify self-efficacy and adaptation.

## Case Study

### Participant

Ann (pseudonym), a 57-year-old Vietnamese woman, sought counselling following her loved one’s sudden death in 2024. After over 30 years of a loving marriage, she experienced shock (“I feel lonely”), sadness, helplessness, anger, and meaninglessness, compounded by physical symptoms like insomnia and fatigue. As a mother of two children living abroad, Ann struggled to balance her grief with caregiving responsibilities. Her Buddhist beliefs, emphasizing fate (“mệnh số”), impermanence, and interconnectedness, were central to her identity, as were her strong community ties in her Vietnamese neighbourhood. Ann consented to share her anonymous story for research, reflecting her desire to support others experiencing loss and aligning with her collectivist values.

### Procedure

Narrative therapy was conducted over seven sessions (August 17, 2024–April 21, 2025) by a licensed therapist trained in narrative approaches. The treatment followed Herman's (2015) three-stage trauma recovery model—safety, remembrance, and mourning, and reconnection—tailored to Ann’s cultural and spiritual context. Techniques included externalization, deconstruction, unique outcome questions, and family dialogue, targeting resilience components such as self-efficacy, adaptation, purpose, and resource utilization (Luthar et al., 2000). Each session honoured Ann’s unique narrative. The therapist ensured cultural sensitivity by incorporating Ann’s Buddhist beliefs and collectivist values, using questions that resonated with her spiritual and social framework.

To reduce redundancy, the following stage descriptions integrate session details without repeating overlapping content between summaries and specific dialogues.

#### Stage 1: Facing Struggle and Externalizing Pain (Sessions 1–2)

**Session 1 (August 17, 2024):** Ann presented with pro-found distress, articulating guilt (“I did not take him to the hospital”), sadness, anger, loneliness, and fear of darkness and isolation. Her Buddhist belief in spiritual continuity allowed her to sense her husband’s enduring love, which she described as a lingering presence that both comforted and pained her.

*Therapist*: “How do you feel at this moment regarding the unexpected passing of your loved one?”

*Client*: “I am afraid to be alone; I cannot accept that my husband is no longer alive. If I had insisted on taking him to the hospital, he might still be alive.”

The client explained that she felt lost, overwhelmed with sorrow, and full of self-blame for the loss of her loved one.

*Client*: “I fear the rain, I fear the darkness, and here I am, truly alone. Even though he is gone, I feel that he sees me crying, feels my pain, my loneliness, my sadness, and my anger. I believe he can understand my heart, even from afar.”

The therapist listened carefully, acknowledging her feelings of fear, isolation, despair, helplessness, guilt, and anger.

*Therapist*: “If you could speak to him now, what would you say?”

This elicited sobs and expressions of anger (“Why did you leave me?”) and guilt, The therapist validated her emotions without judgment (Morgan, 2000). Open-ended questions, such as “How do you feel when you say he has gone?” encouraged Ann to voice her turmoil, thereby initiating self-efficacy.

The client named her guilt “the weight of blame,” a metaphor that resonated with her cultural tendency toward self-criticism (Carr, 1998). Her Buddhist perspective, viewing suffering as part of life’s impermanence, provided a cultural anchor, allowing her to frame her pain within a spiritual context.

*Outcome*: Ann began externalizing guilt, reducing its intensity, and laying the groundwork for resilience by acknowledging her emotions as valid.

**Session 2 (August 30, 2024)**: This was a family session which included Ann’s children, who joined the conversation, fostering mutual understanding (Freedman & Combs, 1996). The question, “What do you need from each other?” prompted Ann to acknowledge her children’s struggles: “I need to understand you, too.” This dialogue was pivotal, as it shifted Ann’s focus from her grief to her role within the family, aligning with collectivist values.

Externalization, using her husband’s image, allowed the family to express both gratitude and regret, thereby strengthening their bonds. For example, Ann’s daughter shared memories of her father’s kindness, prompting Ann to reflect on the shared moments of joy they had.

Reflective questioning (“After hearing your children, what do you need from them?”) enhanced Ann’s self-efficacy by reinforcing her role as a supportive mother, initiating adaptation to new family dynamics (Walsh, 2007).

*Outcome*: Ann’s focus shifted toward family resilience, fostering a sense of interconnectedness and agency within her collectivist cultural framework.

#### Stage 2: Exploring New Meanings and Elaborating Efforts (Sessions 3–4)

**Session 3 (September 10, 2024):** Ann reiterated her pain but showed openness to hope, supported by her children and neighbours. She described small moments of connection, such as neighbours bringing her meals, which resonated with her collectivist values. The therapist asked, “What is your greatest source of hope?” Ann identified her family and community, highlighting resource utilization (Morgan, 2000).

Deconstruction explored her guilt through Buddhist beliefs about fate, helping her view her husband’s death as part of “mệnh số” (fate), beyond her control. The therapist asked, “How does your belief in fate shape the way you see your husband’s passing?” Ann responded, “I think it was his time, but I still feel I failed him.” This dialogue allowed Ann to contextualize her guilt within a spiritual framework, reducing self-blame.

Relaxation techniques, including facial acupressure and soothing music, addressed her insomnia and fatigue, supporting physical and emotional adaptation (Kowalski & Bondmass, 2007).

*Outcome*: Ann’s identification of resources and engagement with Buddhist practices strengthened her ability to oscillate between grief and responsibilities, aligning with Stroebe & Schut's (1999) Dual Process Model.

**Session 4 (September 17, 2024):** Ann reported finding comfort in the Inner Space community program, a local initiative for bereaved women, where she connected with others sharing similar experiences. This engagement was a turning point, as it provided a safe space to share her story and feel understood.

The miracle question, “If your husband were here, what would he want for you?” fostered a sense of purpose, with Ann stating, “He would want me to be happy and take care of our children” (Hedtke, 2014).

Unique outcome questions (“What joy has supported you?”) prompted Ann to reflect on moments of connection at Inner Space, reinforcing self-efficacy. An internal dialogue (“Where would you place him in your life now?”) led Ann to say, “In my heart, always,” marking a significant adaptive shift (Neimeyer, 2001). This shift reflected her ability to integrate her husband’s memory into her identity without being overwhelmed by grief.

*Outcome*: Ann’s narrative began to incorporate hope and purpose, reflecting her growing ability to re-author her story within her cultural and spiritual context.

#### Stage 3: Re-authoring for Resilience (Sessions 5-7)

**Session 5 (October 8, 2024):** Ann reported learning to “close it with respect and love,” choosing to hold her husband’s memory in her heart rather than being consumed by grief. She expressed a desire to move forward for her children: “I need to continue what my husband left unfinished.”

Her engagement with Inner Space allowed her to share her story, inspiring other women and fostering a sense of empathy and community. Ann acknowledged, “It is acceptable to have a problem; it is okay to ask for help,” reflecting a cultural shift from stoicism to openness, which is significant in a collectivist context where seeking help can be stigmatized (Ungar, 2011).

Reflective questions (“What activities have helped you most?”) highlighted Inner Space’s role in fostering purpose (Morgan, 2000). Ann described how sharing her story at Inner Space made her feel “less alone,” reinforcing her sense of community. Outcome: Ann’s narrative re-authoring emphasized clarity and openness, supporting post-traumatic growth (Lichtenthal et al., 2010).

**Session 6 (October 19, 2024):** Unique outcome questions (“What is contributed to this change in how you feel?”) prompted Ann to credit Inner Space and her role in helping others, reinforcing self-efficacy. She described teaching yoga to other women at Inner Space, which gave her a sense of purpose and agency.

Re-authoring clarified “packing it up” as keeping her husband in her heart while moving forward, a metaphor that resonated with her Buddhist belief in holding onto love without attachment (Neimeyer et al., 2006). Ann expressed gratitude for her husband’s legacy—his love for their children and humanity—and consented to share her story for research, reflecting her commitment to supporting others.

*Outcome*: Ann’s narrative solidified around purpose and resilience, with community engagement playing a central role.

**Session 7 (April 21, 2025):** Ann’s demeanour showed marked improvement, with a brighter expression and a renewed engagement in life, such as wearing a colourful dress, which she noted was a tribute to her husband’s love for her vibrancy. She revisited her grief, stating, “I am scared, I feel suffering, I cannot accept it.”

The therapist’s questions, such as “Do you feel he understands your heart right now?” helped Ann articulate her husband’s enduring presence: “I had a delighted life with him.” Reflecting on Buddhist concepts of fate, Ann said, “People have their fate, don’t they? I did everything I could.” This realization, rooted in “mệnh số,” allowed her to reframe her guilt as part of a larger cosmic order.

Her final statement, “I have decided to pack up the pain… keep it in my heart, and move forward with my children. I am grateful to him; he left me a sky of love, two wonderful children, his legacy of love for humanity,” reflected a forward-looking narrative (Hedtke & Winslade, 2016). The therapist affirmed this shift, reinforcing Ann’s resilience and purpose.

*Outcome*: Ann fully integrated her husband’s memory into a new narrative, balancing grief with a commitment to her family and community.

### Results

By Session 7, Ann had transformed her grief narrative from one dominated by guilt and helplessness to one of resilience, evidenced by improved emotional well-being, stronger family bonds, and active community engagement through Inner Space and yoga. Her final statement, “I have decided to pack up the pain… not to throw it away, but to keep it in my heart… I will carry his love with me and move forward with our children,” encapsulates the integration of self-efficacy (confidence in coping), adaptation (new life narrative), purpose (honoring her husband’s legacy), and resource utilization (family, community).

Her Buddhist beliefs, particularly “mệnh số,” played a pivotal role in reducing self-blame, aligning with collectivist values that prioritize family and community over individual guilt (Ungar, 2011). Her participation in Inner Space, where she shared her story and inspired others, underscored her purpose and reflected post-traumatic growth (Cacciatore et al., 2021).

The three-stage trauma recovery model (Herman, 2015) structured her progress: Stage 1 established safety for emotional expression. Stage 2 facilitated meaning-making through resource identification and deconstruction, while Stage 3 marked her reconnection with life through her roles within family and community. Ann’s consent to share her story for research further highlighted her purpose, contributing to collective healing within her cultural context.

## Discussion

Our study took place in a field of psychotherapy that we are familiar with and are also participating in. This calls for discretion and critical self-reflection when it comes to interpretations, preconceived notions and values, to maintain a sensitivity to the studied phenomena. In accordance with Interpretive Phenomenological Analysis (Smith et al, 2019), we started from the basic assumption that we live in a world of experiences, and that we (including our informants) gain knowledge through active interpretations of our experiences.

### Self-efficacy: Building confidence

Self-efficacy, or the belief in one’s ability to cope, is critical for grief recovery (Bandura, 1997). However, cultural norms that value stoicism may hinder the acknowledgment of strengths, requiring culturally sensitive approaches (Ungar, 2011).

Examples from the treatment, presented chronologically: In Session 1, externalization (projecting her husband’s image) enabled Ann to express guilt without self-identification, initiating self-efficacy (Walsh, 2007). In Session 2, reflective questioning enhanced Ann’s self-efficacy by reinforcing her role as a supportive mother. In Session 3, unique outcome questions helped Ann identify family and community as resources, building confidence. In Session 4, the miracle question fostered purpose, further supporting self-efficacy. In Session 5, Ann acknowledged “It is acceptable to have a problem; it is okay to ask for help,” reflecting growing self-efficacy. In Session 6, crediting Inner Space reinforced her ability to help others. By Session 7, Ann recalled moments of strength, such as “I am trying so hard to keep going,” solidifying her confidence.

### Adaptation: Re-authoring to new realities

Adaptation involves integrating loss into a new life narrative (Walsh, 2006). Neimeyer (2001) emphasizes narrative reconstruction as a means of meaning-making, while Hedtke and Winslade (2016) note that re-authoring fosters hope. Challenges include resistance due to guilt (Angus & Hardtke, 1994), but longitudinal studies on sustainability are limited (Payne, 2006).

Examples from the treatment, presented chronologically: In Session 2, family dialogue initiated adaptation to new family dynamics (Walsh, 2007). In Session 3, relaxation techniques supported physical and emotional adaptation (Kowalski & Bondmass, 2007). By Session 4, Ann shifted to “I carry his love for my children,” reflecting adaptation. In Session 5, narrative re-authoring emphasized openness to new realities. In Session 6, “packing it up” as keeping her husband in her heart marked adaptive progress. In Session 7, Ann’s statement “I have decided to pack up the pain… and move forward” fully demonstrated re-authoring to new realities.

### Purpose: Finding purpose and meaning

The experience of purpose and meaning is often disrupted by grief (Neimeyer et al., 2014). Narrative therapy can restore purpose by connecting loss to legacy through questions. Hedtke (2014) highlights narrative therapy’s role in hopeful stories, and Lichtenthal et al. (2010) found that narrative interventions promote post-traumatic growth. Research gaps exist regarding the long-term maintenance of purpose, especially among older caregivers.

Examples from the treatment, presented chronologically: In Session 4, the miracle question led Ann to affirm her husband’s desire for her happiness, initiating purpose. In Session 5, sharing her story at Inner Space fostered purpose through helping others. In Session 6, teaching yoga gave Ann a sense of agency and purpose. By Session 7, Ann articulated gratitude for her husband’s legacy, linking purpose to family and community involvement.

### Resource utilization: Leveraging supports

Resource utilization involves accessing internal (e.g., perseverance) and external (e.g., community) supports (Ungar & Theron, 2019). Questions like “What has supported you?” amplified these (Morgan, 2000). Walsh (2006) emphasizes the importance of resource reconnection, but few studies have explored culturally specific resources in collectivist settings (Hedtke & Winslade, 2016).

Examples from the treatment, presented chronologically: In Session 3, Ann identified family and neighbours as resources (Morgan, 2000). In Session 4, engagement with Inner Space provided community support. In Session 5, reflective questions highlighted community resources. In Session 6, crediting Inner Space reinforced resource use. By Session 7, Ann fully leveraged family, community, and Buddhist beliefs for ongoing support.

### Cultural context in grief processing

The cultural context is pivotal in Ann’s case. Vietnam’s collectivist culture emphasizes family and community roles, placing significant expectations on individuals to fulfill familial duties (Ungar, 2011). This can intensify feelings of guilt, as seen in Ann’s self-blame for not preventing her husband’s death. However, Buddhist beliefs about fate (“mệnh số”) and impermanence provided a spiritual framework for processing loss, helping Ann contextualize her husband’s death as part of a larger cosmic order (Ungar, 2011). These beliefs reduced self-blame by situating her experience within a cultural narrative of acceptance and interconnectedness. The collectivist emphasis on community also facilitated Ann’s engagement with the Inner Space program, where she found solace and purpose among other bereaved women.

Gaps in the literature persist regarding the application of narrative therapy in non-Western, collectivist settings and the sustainability of resilience outcomes (Payne, 2006). This case study addresses these gaps by illustrating how narrative treatment, tailored to Ann’s cultural and spiritual context, facilitated resilience through a person-oriented lens.

### Theoretical gaps and contributions

While narrative therapy has been widely studied in Western contexts, its application in non-Western, collectivist cultures remains underexplored (Payne, 2006). Ann’s case highlights the importance of cultural sensitivity in therapeutic interventions, particularly in integrating spiritual beliefs like Buddhism into grief processing. The literature also lacks longitudinal studies on the sustainability of narrative therapy’s outcomes in grief counselling (Payne, 2006). This case study contributes to the literature by documenting Ann’s journey over seven sessions, offering insights into the process of resilience-building and suggesting directions for future research, such as exploring long-term outcomes in diverse cultural settings.

This case study illustrates the capacity of narrative therapy to promote resilience within a person-centred, culturally sensitive framework. Externalization reduced Ann’s self-blame by reframing guilt as an external narrative, allowing her to engage with her emotions without self-identification (Carr, 1998). Deconstruction, grounded in Buddhist concepts of fate, helped her contextualize her husband’s death, aligning with collectivist values that emphasize interconnectedness (Ungar, 2011). Unique outcome questions unearthed moments of strength, such as her roles as a mother and community member, which fostered alternative narratives of hope and agency (Morgan, 2000). The therapy’s alignment with Ann’s cultural and spiritual context enhanced its effectiveness, addressing gaps in the literature regarding non-Western applications of narrative therapy (Payne, 2006).

The person-oriented approach of this study emphasizes Ann’s unique experiences and cultural narratives, aligning with a focus on individualized care (Wisdom et al., 2008). Ann’s journey reflects Stroebe & Schut’s (1999) Dual Process Model, with narrative therapy facilitating both emotional processing and practical engagement. The integration of Buddhist beliefs, particularly “mệnh số,” was crucial in reducing self-blame, as it provided a spiritual framework for accepting the unpredictability of loss. Similarly, her collectivist values facilitated her engagement with Inner Space, reinforcing community as a source of resilience (Cacciatore et al., 2021). The therapy’s success highlights the importance of tailoring interventions to the client’s cultural and spiritual context, particularly in non-Western settings where collectivist values shape grief experiences.

Limitations include the single-case design, which restricts generalizability, and the dependence on therapist skill in crafting culturally attuned questions (Monk et al., 1997). The effectiveness of narrative therapy relies on the therapist’s ability to navigate cultural nuances, such as Ann’s Buddhist beliefs, which may not be replicable without specialized training. Additionally, the study’s focus on short-term outcomes leaves questions about the sustainability of Ann’s resilience unanswered (Payne, 2006). Future research should explore longitudinal outcomes to assess the durability of narrative therapy’s effects in grief counselling, particularly in collectivist cultures. Further studies could also investigate the applicability of narrative therapy across diverse non-Western populations, addressing gaps in cultural specificity (Ungar, 2011).

### Recommendations

Based on Ann’s case, the following recommendations are proposed for clinicians using narrative therapy to support clients experiencing sudden loss:

1. *Incorporate early externalization*: Clinicians should introduce externalization in initial sessions to separate clients from overwhelming emotions like guilt. Naming emotions (e.g., “the weight of blame”) fosters agency by distancing clients from problem-saturated narratives, allowing space for self-compassion (Carr, 1998).
2. *Leverage cultural narratives*: In culturally diverse settings like Vietnam, clinicians should explore beliefs such as “mệnh số” to contextualize loss. This approach aligns with collectivist values, reducing self-blame by situating events within broader spiritual or cultural frameworks (Ungar, 2011).
3. *Focus on unique outcome questions*: Therapists should use questions like “When did you feel less over-whelmed?” to highlight moments of strength and connection, constructing alternative narratives that emphasize resilience and meaning (Morgan, 2000).
4. *Facilitate re-authoring for resilience*: Clinicians should guide clients toward re-authoring their stories, encouraging forward movement while honouring the loss. For example, helping clients “pack up” grief in alignment with their values (e.g., family, legacy) promotes post-traumatic growth (Lichtenthal et al., 2010).
5. *Encourage community engagement*: Leveraging community resources, such as programs like Inner Space, strengthens social bonds and purpose, particularly in collectivist cultures where community ties are central (Cacciatore et al., 2021).
6. *Longitudinal follow-up:* Given the single-case design, clinicians should conduct follow-up sessions to assess the sustainability of resilience. Monthly check-ins for six months are recommended to support ongoing adjustment and commitment to family and legacy (Payne, 2006).
7. *Training in culturally sensitive narrative therapy*: Clinicians should receive training in narrative techniques, particularly externalization and re-authoring, with an emphasis on cultural sensitivity to enhance outcomes in diverse populations (Monk et al., 1997).

## Action editor

Lars-Gunnar Lundh served as action editor for this article.

## Open access

This article is distributed under the terms of the Creative Commons Attribution 4.0 International License (http://creativecommons.org/licenses/by/4.0/), which permits unrestricted use, distribution, and reproduction in any medium, provided you give appropriate credit to the original author(s) and the source, provide a link to the Creative Commons license, and indicate if changes were made.

## References

[cit0001] Angus, L., & Hardtke, K. (1994). Narrative processes in psychotherapy. *Canadian Psychology*, 35 (2), 190203. 10.1037/0708-5591.35.2.190

[cit0002] Bandura, A. (1997). *Self-efficacy: The exercise of control*. Macmillan.

[cit0003] Blackledge, J. T., & Hayes, S. C. (2001). Emotion regulation in Acceptance and Commitment Therapy. *Journal of Clinical Psychology*, 57(2), 243-255. 11180150 10.1002/1097-4679(200102)57:2<243::aid-jclp9>3.0.co;2-x

[cit0004] Bonanno, G. A. (2004). Loss, trauma, and human resilience. *American Psychologist*, 59(1), 20–28. 10.1037/0003-066X.59.1.20 14736317

[cit0005] Cacciatore, J., et al. (2021). The role of social connection in grief. *Death Studies*, 45(1), 110. 10.1080/07481187.2020.173696731122149

[cit0006] Carr, A. (1998). Michael White's narrative therapy. *Contemporary Family Therapy*, 20 (4), 485503. 10.1023/A:1021680116584

[cit0007] Connor, K. M., & Davidson, J. R. (2003). Development of a new resilience scale: The Connor-Davidson Resilience Scale (CD-RISC). *Depression and Anxiety*, 18(2), 7682. 10.1002/da.10113 12964174

[cit0008] Freedman, J., & Combs, G. (1996). *Narrative therapy: The social construction of preferred realities*. Norton.

[cit0009] Hedtke, L. (2014). Creating stories of hope: A narrative approach to illness, death, and grief. *Australian and New Zealand Journal of Family Therapy*, 35(1), 417. 10.1002/anzf.1040

[cit0010] Hedtke, L., & Winslade, J. (2016). *The crafting of grief: Constructing aesthetic responses to loss*. Routledge.

[cit0011] Herman, J. L. (2015). *Trauma and recovery: The aftermath of violence*. Basic Books.

[cit0012] Keyes, K. M., et al. (2014). The burden of loss: Unexpected death of a loved one and psychiatric disorders. *American Journal of Psychiatry*, 171(8), 864871. 10.1176/appi.ajp.2014.13081132 PMC4119479 24832609

[cit0013] Khasanah, Z., & Habsy, B. A. (2025). Theory and application of narrative counselling. *Al-Ihath Jurnal Bimbingan Dan Konseling Islam*, 5(1), 27–34. 10.53915/jbki.v5i1.570

[cit0014] Knopf, H. T., & Swick, K. J. (2008). Using our understanding of families to strengthen family involvement. *Early Childhood Education Journal*, 35(5), 419-427.

[cit0015] Kowalski, S. D., & Bondmass, M. D. (2007). Physiological and psychological symptoms of grief in widows. *Research in Nursing & Health*, 31(1), 2330. 10.1002/nur.20228 18161825

[cit0016] Kübler-Ross, E. (1973). *On death and dying*. Routledge.

[cit0017] Lichtenthal, W. G., et al. (2010). Meaning-making in cancer survivors: A narrative approach. *Journal of Loss and Trauma*, 15(5), 419–435. 10.1080/15325024.2010.507658

[cit0018] Luthar, S. S., et al. (2000). The construct of resilience: A critical evaluation and guidelines for future work. *Child Development*, 71(3), 543562. 10.1111/1467-8624.00164 PMC1885202 10953923

[cit0019] Monk, G., et al. (1997). *Narrative therapy in practice: The archaeology of hope*. Jossey Bass.

[cit0020] Morgan, A. (2000). *What is narrative therapy? An easy-to-read introduction*. Dulwich Centre Publications.

[cit0021] Neimeyer, R. A. (2001). *Meaning reconstruction and the experience of loss*. American Psychological Association.

[cit0022] Neimeyer, R. A., et al. (2006). Meaning reconstruction in the wake of loss: Evolution of a research program. *Journal of Loss and Trauma*, 11(5), 353369. 10.1080/15325020600691797

[cit0023] O'Brien, M. R., et al. (2016). Bereavement support and prolonged grief disorder among carers. *British Journal of Neuroscience Nursing*, 12(6), 268276. 10.12968/bjnn.2016.12.6.268

[cit0024] Payne, M. (2006). *Narrative therapy: An introduction for counsellors*. Sage.

[cit0025] Stroebe, M., & Schut, H. (1999). The dual process model of coping with bereavement: Rationale and description. *Death Studies*, 23(3), 197224. 10.1080/074811899201046 10848151

[cit0026] Tchalova, K., & Eisenberger, N. (2015). How the brain feels the hurt of heartbreak. *Brain Mapping: An Encyclopedic Reference* 10.1016/B978-0-12-397025-1.00144-5

[cit0027] Ungar, M. (2011). The social ecology of resilience. *American Journal of Orthopsychiatry*, 81(1), 1–17. 10.1111/j.1939-0025.2010.01067.x 21219271

[cit0028] Ungar, M., & Theron, L. (2019). Resilience and mental health: How multisystemic processes contribute to positive outcomes. *The Lancet Psychiatry*, 7(5), 441–448. 10.1016/S2215-0366(19)30434-1 31806473

[cit0029] Walsh, F. (2007). Traumatic loss and major disasters: Strengthening family and community resilience. *Family Process*, 46(2), 207227. 10.1111/j.1545-5300.2007.00205. 17593886

[cit0030] White, M., & Epston, D. (1990). *Narrative means to therapeutic ends*. Norton.

[cit0031] Worden, J. W. (2018). *Grief counseling and grief therapy: A handbook for the mental health practitioner*. Springer Publishing Company.

